# The emergence of Pax7-expressing muscle stem cells during vertebrate head muscle development

**DOI:** 10.3389/fnagi.2015.00062

**Published:** 2015-05-19

**Authors:** Julia Meireles Nogueira, Katarzyna Hawrot, Colin Sharpe, Anna Noble, William M. Wood, Erika C. Jorge, David J. Goldhamer, Gabrielle Kardon, Susanne Dietrich

**Affiliations:** ^1^School of Pharmacy and Biomedical Sciences, Institute for Biomedical and Biomolecular Science, University of PortsmouthPortsmouth, UK; ^2^Departamento de Morfologia, Instituto de Ciências Biológicas, Universidade Federal de Minas GeraisBelo Horizonte, Brazil; ^3^School of Biological Sciences, Institute for Biomedical and Biomolecular Science, University of PortsmouthPortsmouth, UK; ^4^European Xenopus Resource Centre, School of Biological Sciences, University of PortsmouthPortsmouth, UK; ^5^Department of Molecular and Cell Biology, University of Connecticut Stem Cell Institute, University of ConnecticutStorrs, CT, USA; ^6^Department of Human Genetics, University of UtahSalt Lake City, UT, USA

**Keywords:** head muscle, muscle stem cells, Pax7, chicken, mouse, Xenopus, zebrafish, vertebrate embryo

## Abstract

*Pax7* expressing muscle stem cells accompany all skeletal muscles in the body and in healthy individuals, efficiently repair muscle after injury. Currently, the *in vitro* manipulation and culture of these cells is still in its infancy, yet muscle stem cells may be the most promising route toward the therapy of muscle diseases such as muscular dystrophies. It is often overlooked that muscular dystrophies affect head and body skeletal muscle differently. Moreover, these muscles develop differently. Specifically, head muscle and its stem cells develop from the non-somitic head mesoderm which also has cardiac competence. To which extent head muscle stem cells retain properties of the early head mesoderm and might even be able to switch between a skeletal muscle and cardiac fate is not known. This is due to the fact that the timing and mechanisms underlying head muscle stem cell development are still obscure. Consequently, it is not clear at which time point one should compare the properties of head mesodermal cells and head muscle stem cells. To shed light on this, we traced the emergence of head muscle stem cells in the key vertebrate models for myogenesis, chicken, mouse, frog and zebrafish, using *Pax7* as key marker. Our study reveals a common theme of head muscle stem cell development that is quite different from the trunk. Unlike trunk muscle stem cells, head muscle stem cells do not have a previous history of *Pax7* expression, instead *Pax7* expression emerges *de-novo*. The cells develop late, and well after the head mesoderm has committed to myogenesis. We propose that this unique mechanism of muscle stem cell development is a legacy of the evolutionary history of the chordate head mesoderm.

## Introduction

Adult skeletal muscle stem cells (satellite cells) accompany contractile muscle fibers and efficiently repair muscle after injury (reviewed in Relaix and Zammit, 2012). It is generally thought that one of the factors contributing to this efficient repair is that skeletal muscle stem cells are tissue-specific stem cells, solely committed to myogenesis. However, in a number of diseases including muscular dystrophies, cancer and HIV/Aids, the ability of muscle stem cells to repair muscle is compromised. Moreover, muscle regeneration declines when we age. This has been ascribed to inflammatory responses, changes to the stem cell niche and changes to the stem cells themselves. Current approaches investigate how these parameters could be targeted to reinstate the full regenerative capacity of muscle.

Overall, skeletal muscle function and repair is much the same in all areas of the body. Therefore, it is often overlooked that muscular dystrophies differentially target muscle groups in the head and in the trunk (reviewed in Emery, 2002). Moreover, head and trunk muscle and their accompanying muscle stem cells have a different developmental history (reviewed in Sambasivan et al., 2011, and see below). This tissue also contributes to the heart, an organ that in amniotes including humans cannot regenerate (reviewed in Garbern and Lee, 2013). Moreover, adult head and trunk muscle stem cells have divergent gene expression, proliferation and differentiation profiles (Sambasivan et al., 2009; Ono et al., 2010; Hebert et al., 2013). Thus, the head mesoderm and the muscle stem cells derived thereof are of great interest to develop both specialized skeletal muscle stem cells and cardiac cells for human therapy.

In the body, skeletal muscles and their accompanying stem cells develop from the segmented paraxial mesoderm, the somites (reviewed in Bryson-Richardson and Currie, 2008; Buckingham and Vincent, 2009; Relaix and Zammit, 2012). Muscles (myotomes) are laid down in waves, and while the first cells differentiate into contractile fibers, more cells are being added on from a dual muscle-dermis-competent precursor pool, stored in a specialized somitic compartment, the dermomyotome. This compartment also provides cells that emigrate into the periphery to provide the limb, hypobranchial/hypopharyngeal/hypoglossal, and in mammals, diaphragm muscles. Importantly, cells in the dermomyotome eventually shed their dermal competence, enter the myotome and become specialized muscle stem cells (Gros et al., 2005; Kassar-Duchossoy et al., 2005; Relaix et al., 2005; Schienda et al., 2006). These cells actively self-renew and provide differentiating cells during fetal and juvenile stages of development, thereby providing the bulk of the adult musculature. Eventually, the stem cells settle underneath the basal lamina of the differentiated muscle fibers and become adult muscle stem cells (satellite cells). In amniotes, these stem cells adopt a quiescent state, only to be activated when injuries occur; in anamniotes, the cells may remain mitotically active and continue to drive muscle growth (Bryson-Richardson and Currie, 2008; Buckingham and Vincent, 2009; Relaix and Zammit, 2012).

In amniotes, somites express the paralogous transcription factors Pax3 and Pax7 as soon as they form; in all jawed vertebrates, these genes continue to be expressed in the dermomyotome, with either Pax3 or both proteins also labeling the migratory muscle precursors (reviewed in Bryson-Richardson and Currie, 2008; Buckingham and Vincent, 2009; Relaix and Zammit, 2012). The genes keep cells in a proliferative state, but are also required to initiate myogenesis, and hence are referred to as premyogenic genes (Collins et al., 2009; Diao et al., 2012; Kawabe et al., 2012). Cells undertaking differentiation then switch on members of the MyoD family of transcription factors, which are crucial for myogenic differentiation (Weintraub et al., 1989). In the somites, *Myf5* and *MyoD* are expressed first and commit cells to myogenesis. In a feed forward mechanism, they activate *Myogenin* which promotes cell cycle exit and entry into terminal differentiation (Penn et al., 2004). *Mrf4* has an early expression phase in the mouse (Summerbell et al., 2002), but in most models, acts mainly during fetal myogenesis (Hinits et al., 2009; Della Gaspera et al., 2012, and Dietrich, unpublished observations).

The *Pax3* and *Pax7* genes arose as a result of the second of two rounds of whole genome duplications that occurred in the ancestors of jawed vertebrates 500 million years ago (Ohno et al., 1968; Holland et al., 1994). In jawless vertebrates, the single *pax3/7* gene is also expressed in dermomyotomal muscle precursors (Kusakabe et al., 2011). Likewise, *pax3/7* expression has also been found in the somites and muscle stem cell-like cells of the cephalochordate Amphioxus (Holland et al., 1999; Somorjai et al., 2012), indicating an ancient role as premyogenic genes. In jawed vertebrates, both genes were subject to subfunctionalisation: cells retaining muscle stem cells properties rely on the presence of *Pax7* rather than *Pax3*, and in the absence of *Pax7* function, the deposition and maintenance of the skeletal muscle stem cell pool is impaired (Seale et al., 2000; Kassar-Duchossoy et al., 2005; Relaix et al., 2006; Lepper et al., 2009; von Maltzahn et al., 2013). Moreover, in anamniote vertebrates such as the axolotl, in which fully differentiated, functional muscle can contribute to regeneration by returning to a stem cell state, or in experimental models where de-differentiation is induced *in vitro*, this occurs concomitant with a reactivation of *pax7* (Kragl et al., 2009; Pajcini et al., 2010). Thus, the *Pax7* gene is accepted as the universal skeletal muscle stem cell marker in jawed vertebrates.

In the head, the muscles that move the eye ball, move the gill arches and in jawed vertebrates, open and close the mouth, are derived from the non-somitic paraxial head mesoderm (Noden, 1983; Couly et al., 1992; Harel et al., 2009; Sambasivan et al., 2009; reviewed in Sambasivan et al., 2011). This tissue does not form segments, and in contrast to the trunk mesoderm, contributes to both, skeletal muscle and the heart. The early head mesoderm does not express the *Pax3* gene and instead, harbors a complement of markers whose expression pattern is established in a step-wise fashion; eventually, the eye and jaw closure muscle anlagen express *Pitx2*, the most posterior eye muscle and muscle anlagen for the jaw and throat (branchiomeric muscles) express *Tbx1*, and all express *Musculin* (*Msc/MyoR*) (Mootoosamy and Dietrich, 2002; Bothe and Dietrich, 2006; Bothe et al., 2011). These transcription factors have overlapping roles. Notably, similar to *Pax3* and *Pax7* in the trunk, they keep cells in an immature state, control their survival and activate *MyoD* family members; once *Mrf* genes are expressed, myogenic differentiation is thought to occur in a similar fashion as in the body (Kitamura et al., 1999; Lu et al., 2002; Kelly et al., 2004; Diehl et al., 2006; Dong et al., 2006; Zacharias et al., 2011; Moncaut et al., 2012; Hebert et al., 2013; Castellanos et al., 2014).

In the adult, head muscle is equipped with muscle stem cells which express *Pax7*, underlining that *Pax7* is the bona fide muscle stem cell marker (Harel et al., 2009; Sambasivan et al., 2009, reviewed in Sambasivan et al., 2011). These stem cells however are not immigrants from the somites. Rather, like the muscle they accompany, they are derived from the head mesoderm itself. In tune with this observation, head muscle stem cells continue to express the early head mesodermal markers. This implies that head muscle stem cells may have retained some of the properties of the early head mesoderm, and may therefore be suited to developing specialized muscle stem cells and cardiac cells for therapy.

To explore the developmental and therapeutic potential of head muscle stem cells, we need to understand when and how these cells are being generated. This is currently not known. The aim of this study therefore is to establish when and where head muscle stem cells emerge, using *Pax7* as lead-marker. In order to understand the basic process common to all jawed vertebrates, we investigated the key models for vertebrate myogenesis, chicken, mouse, frog (sarcopterygians), and zebrafish (an actinopterygian). Our work shows that unequivocally, *Pax7* expressing cells arise late in head muscle development, well after the onset of *Myf5* and *MyoD*. Importantly, the cells arise from *MyoD* expressing precursor cells, and we propose that head mesodermal cells have to commit to myogenesis before being able to become a muscle stem cell.

## Materials and methods

### Culture and staging of embryos

#### Chicken embryos

Fertilised chicken eggs (Henry Stewart Ltd, Norfolk) were incubated in a humidified atmosphere at 38.5°C and staged according to Hamburger and Hamilton (1951). Embryos were harvested in 4% PFA.

#### Mouse embryos

Wildtype mice were provided by the Animals Resource Centre at the University of Portsmouth. Transgenic mouse driver lines carrying the improved *Cre* gene introduced into the *Pax7* or the *MyoD* locus and reporter lines carrying a Cre-activateable *LacZ, GFP*, or *YFP* gene in the *Rosa26* locus are described in Hutcheson et al. (2009), Kanisicak et al. (2009), and Wood et al. (2013) and were provided by the Kardon and Goldhamer laboratories. Mice were mated overnight; the appearance of a vaginal plug the next morning was taken as day 0.5 of development (E0.5). Pregnant females were sacrificed by cervical dislocation and the embryos were fixed in 4% PFA.

#### Xenopus embryos

Adult J-strain and cardiac actin:GFP transgenic *Xenopus laevis* frogs were maintained in the European Xenopus Resource Centre (EXRC) at the University of Portsmouth at 18°C in a 14 h light 10 h dark cycle and fed 5 days each week using high protein trout pellets. Embryos were generated as described in Guille (1999), then dejellied in 2% cystein-HCl (pH 8.0), grown at 18–23°C in 0.1 × MBS (Gurdon, 1977), staged according to Nieuwkoop and Faber (1994) and harvested in MEMFA (Harland, 1991).

#### Zebrafish embryos

Zebrafish embryos were provided by the INCT de Medicina Molecular, Faculdade de Medicina, Universidade Federal de Minas Gerais. Breeding zebrafish (*Danio rerio*) were maintained at 28°C on a 14 h light/10 h dark cycle. Embryos were obtained by natural spawning, grown in egg water (0.3 gl/l Instant Ocean Salt, 1 mg l/l Methylene Blue) at 28°C and staged according to Kimmel et al. (1995). To prevent pigment formation, embryos post-24 hpf were raised in 0.2 mM 1-phenyl-2-thiourea (PTU, Sigma). Embryos were harvested in 4% PFA.

### Whole mount *in situ* hybridisation, immunohistochemistry, beta galactosidase staining, and sectioning

In chicken and mouse, whole mount *in situ* hybridisation, double *in situ* hybridisation, antibody staining, *in situ* hybridisation followed by antibody staining and vibratome sectioning was carried out as described by Dietrich et al. (1997, 1998, 1999), Mootoosamy and Dietrich (2002), Alvares et al. (2003), and Lours and Dietrich (2005). *In situ* hybridisation and antibody staining in Xenopus followed the protocols by Harland (1991) and Baker et al. (1995); for zebrafish the protocols by Thisse and Thisse (2008) were used. Beta galactosidase staining and antibody staining on cryosections was performed according to Hutcheson et al. (2009), a heat-induced epitope retrieval in 1.8 mM Citric Acid, 8.2 mM Sodium Citrate and signal amplification using the Streptavidin system was used for Pax7. Probes and antibodies are detailed in the table below (Tables 1A–E).

**Table 1A:** **Chicken ISH probes**.

**Gene**	**Source**	**Fragment size (base pairs)**
Pax7	gift from P. Gruss (Goulding et al., 1993)	582
Pitx2	gift from S. Noji (Yoshioka et al., 1998)	800
Alx4	gift from T. Ogura (Takahashi et al., 1998)	1245
MyoR = Msc	own clone (von Scheven et al., 2006)	550
Capsulin	own clone (von Scheven et al., 2006)	600
Tbx1	gift from D. Srivastava (Garg et al., 2001)	380
Myf5	open reading frame, synthesized and cloned into pMK-RQ	785
MyoD	open reading frame, synthesized and cloned into pMK-RQ	909
MyoG	open reading frame, synthesized and cloned into pMK-RQ	694
Mrf4	open reading frame, synthesized and cloned into pMK-RQ	738
Troponin I 1 (Tnni 1)	RT-PCR fragment obtained from E4 cDNA using the primers F2: 5′-AGCAGCTCCCAGGAGATCAG-3′; R2 T7: 5′-TAATACGACTCACTATAGGGAGA-CATGCAGCTGCATGGGCAC-3′ The fragment was verified by sequencing.	921
Cdh4 = R-Cadherin	gift from C. Redies (unpublished PRC fragment)	900
Pax3	gift from P. Gruss (Goulding et al., 1993)	660
Paraxis	gift from E. Olson(Šošic et al., 1997)	717
Six1	gift from C. Tabin (Heanue et al., 1999)	700
Eya1	gift from A. Streit (Christophorou et al., 2009)	1000

**Table 1B:** **Mouse ISH probes**.

**Gene**	**Source**	**Fragment size (base pairs)**
Pax7	gift from P. Gruss (Jostes et al., 1991)	900
Msc = MyoR	gift from R. Kelly (Kelly et al., 2004)	542
Myf5	gift from T. Braun (Braun et al., 1989)	310
MyoD	gift from T. Braun (Braun et al., 1989)	1785
MyoG	gift from T. Braun (Braun et al., 1989)	290

**Table 1C:** ***Xenopus tropicalis* (Xt) and *Xenopus laevis* (Xl) ISH probes**.

**Gene**	**Primers/Subcloning**	**Fragment size (base pairs)**
Xt pax7	F: 5′-AAGCAGGCAGGAGCCAATCA-3′; R-T7: 5′-TAATACGACTCACTATAGGGAGA-ATGGACAGGTCTCAGAAGATG-3′	804
Separate Xl pax7a and 7b probes	RT-PCR Fragments obtained with the above F and R primers and cloned into pGEMT Easy.	804
Xt msc = myor	F: 5′-GGATCTGTGAGTGACACTGAG-3′; RT7: 5′-TAATACGACTCACTATAGGGAGA-AGGTAGAGAGGTGATGTTCTAG-3′	550
Xt myf5	F: 5′-AGAACAGGTAGAAAACTACTACA-3′; R T7:5′-TAATACGACTCACTATAGGGAGA-AATACAAAATGCAGCCAAGTAGA-3′	531
Xt myod	F: 5′-CCTGCRGCTCCAGGAGAAG-3′; R T7: 5′-TAATACGACTCACTATAGGGAGA-AAGTTTCCTTTGGCCTCAGG-3′	587
Xt myog	F: 5′-CAGACCAAAGGTTTTATGACAA-3′; R T7: 5′-TAATACGACTCACTATAGGGAGA-AATGCATATTTGTCTATGATGG-3′	880
Xt mrf4	F: 5′-GCACAGTTTGGATCAGCAGG-3′; R T7: 5′-TAATACGACTCACTATAGGGAGA-TTCCAACACTGTCCATAATTAC-3′	596
Xt desmin	Degenerate primers were used. F: 5′-TCTGCACTCAGTTTYAGAGAA-3′; R T7: 5′-TAATACGACTCACTATAGGGAGA-CATATSTAAGMGAATYAATGGG-3′; M = A + C; Y = C + T; S = G + C	470

*All cDNA fragments were obtained by RT-PCR using the primers below. To generate templates for probe synthesis, either the binding site for the T7 RNA polymerase was introduced with the reverse PCR primer, or fragments were subcloned. The identity of PCR fragments or clones was confirmed by sequencing*.

**Table 1D:** **Zebrafish ISH probes**.

**Gene**	**Source**	**Fragment size (base pairs)**
pax7a isoform 1	gift from A. Fjose (Seo et al., 1998)	2000
myod1	RT-PCR Fragment obtained with F: 5′-TTCTACGACGACCCTTGCTT-3′; R: 5′-GGATTCGCCTTTTTCTGCT-3′; cloned into pGEMT Easy and sequenced	858

**Table 1E:** **Antibodies**.

	**Source**	**Dilution**
**PRIMARY ANTIBODIES**		
Rabbit IgG anti-GFP	Life technologies	1:1000
mouse IgG2b anti-sarcomeric Myosin (MF20)	Developmental Studies Hybridoma Bank	1:200
Mouse IgG1 anti-embryonic skeletal muscle Myosin (F1.652)	Developmental Studies Hybridoma Bank	1:10
Mouse IgG1 anti-slow skeletal muscle Myosin (NOQ7.5 4D)	Sigma	1:4000
Mouse IgG1 anti-fast skeletal muscle Myosin (My32)	Sigma	1:1000
mouse anti-NFM 160kd (RMO 270)	Invitrogen	1:2000
mouse IgG1 anti-Pax7	Developmental Studies Hybridoma Bank	1:25
**SECONDARY ANTIBODIES**		
alkaline phosphatase conjugated sheep anti-Digoxygenin, Fab fragments	Roche	1:2000
horse radish peroxidase (HRP) conjugated goat anti-mouse IgG + IgM (H + L)	Jackson Immuno	1:500
Alexa fluor 594 conjugated goat anti-mouse IgG + IgM (H + L)	Jackson Immuno	1:200
Alexa fluor 488 conjugated donkey anti-rabbit IgG (H + L)	Jackson Immuno	1:200
Biotin conjugated goat anti-mouse IgG1; developed with Streptavidin conjugated Alexa Fluor 594	Jackson Immuno	1:200

### Photomicroscopy

Whole embryos were cleared in 80% glycerol/PBS or, when fluorescent antibodies had been used, in 2.50 mg/ml 1,4-diazabicyclo[2.2.2]octane (DABCO) in 90% glycerol/ PBS. Vibratiome sections were mounted with glycerol, cryosections with Fluoromount (Sigma). Embryos and sections were photographed on a Zeiss Axioskop, using fluorescence or Nomarski optics. Sections in **Figure 9** were photographed using a Zeiss LSM710 confocal miscroscope.

### Research ethics

The work was has been approved by the University of Portsmouth Ethical Review Committee (AWERB No14005) and follows the jurisdiction of the Animals (Scientific Procedures) Act. The work involving Pax7-Cre, MyoDiCre, Rosa26-lacZ, and Rosa26-GFP mouse lines and the cardiac actin; GFP frog line is covered by personal licenses to G. Kardon, D. Goldhamer, and M. Guille.

## Results

### Emergence of Pax7 expressing myogenic cells in chicken craniofacial muscles

#### Time course of Pax7 expression

**Figure 1:**
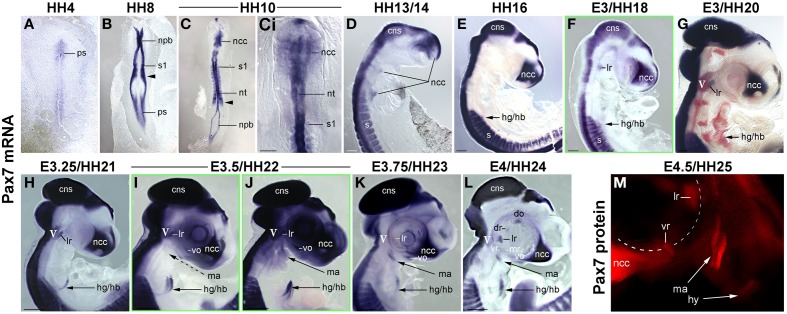
**Time course of *Pax7* expression in the chicken embryo; stages of development are indicated at the top of the panel. (A–L)** Expression of *Pax7* mRNA with (A-Ci) dorsal views, anterior to the top, **(D–M)** lateral views of the right side of embryos, anterior to the top, dorsal to the left. In **(G)**, cranial ganglia are revealed by *Isl1* expression (red staining). In **(L)**, the eye was removed after staining. **(M)** Expression of Pax7 protein; lateral view of the left side of an embryo, anterior to the top, dorsal to the right, the eye has been removed before staining. The onset of *Pax7* expression in craniofacial muscle anlagen is demarcated by green frames to the respective image. Throughout the first 5 days of development, *Pax7* is well detectable in the central nervous system, cranial neural crest cells and the somites and somite-derived muscle precursors/embryonic muscle stem cells. In muscle anlagen derived from the paraxial head mesoderm, expression is first seen at stage HH18 in the lateral rectus eye muscle anlage, the only head muscle to express some trunk markers. In the other head mesoderm derived muscles, *Pax7* mRNA can be detected in strongly stained specimen at stage 22; expression becomes more robust at stages 23–24 and is followed by protein expression at HH25. Abbreviations: cns, central nervous system; do, dorsal oblique eye muscle anlage; dr, dorsal rectus eye muscle anlage; hg/hb, hypoglossal/hypobranchial muscle anlage; lr, lateral rectus eye muscle anlage; ma, mandibular arch muscle anlage (jaw closure muscles); mr, medial rectus eye muscle anlage; ncc, neural crest cells; npb, neural plate border; nt, neural tube; ps, primitive streak; s/s1, somite/ somite 1; vo, ventral oblique eye muscle anlage; vr, ventral rectus eye muscle anlage; V, 5th cranial ganglion (trigeminal ganglion).

**Figure 2:**
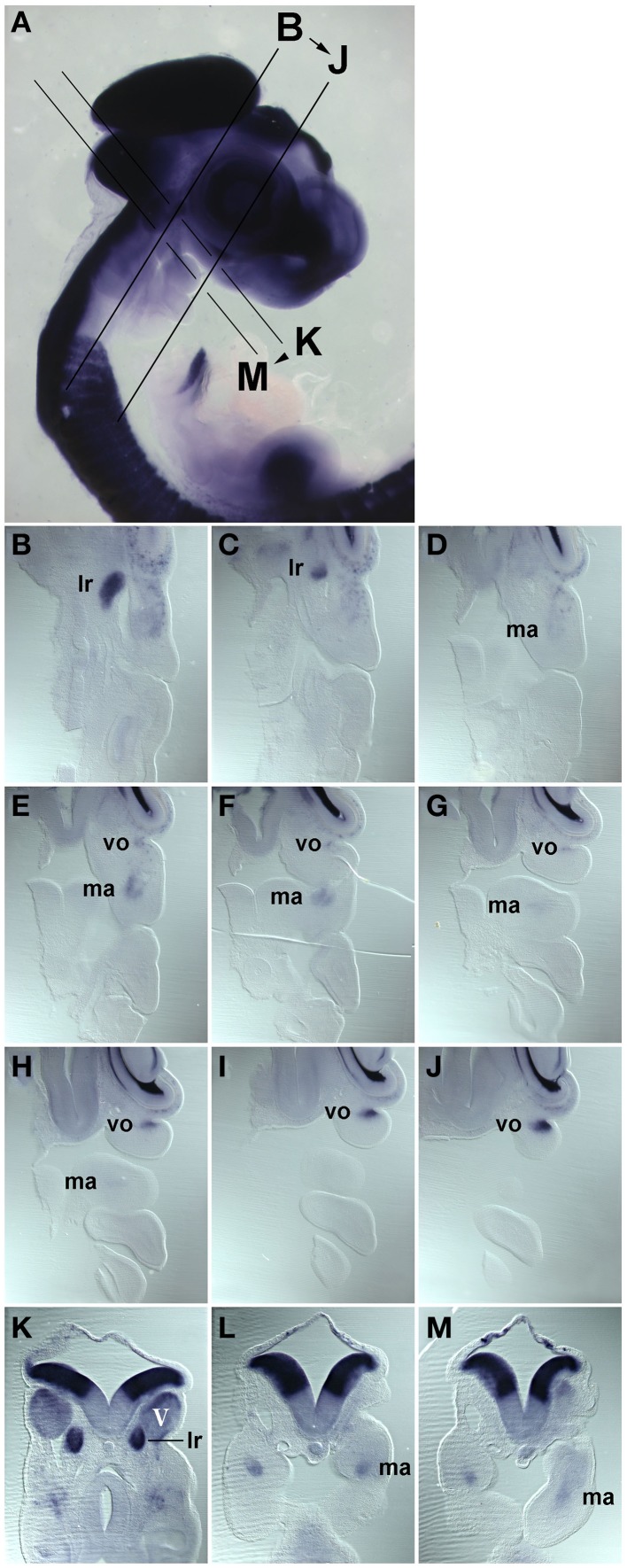
**Series of frontal (B–J) and cross (K–M) sections of a HH22 chicken head, stained for the expression of *Pax7* mRNA, the plane and order of sections in indicated in (A). (B–J)** anterior to the top, lateral to the right; **(K–M)** dorsal to the top. Abbreviations as in Figure 1. The position of the *Pax7* signals beneath the trigeminal ganglion, beneath the eye and in the core of the mandibular arch confirms that these are expression domains associated with developing head muscles.

Muscle stem cells have the ability to self renew and generate differentiating daughter cells, and this ability is linked to the expression and function of *Pax7* (reviewed in Bryson-Richardson and Currie, 2008; Buckingham and Vincent, 2009; Relaix and Zammit, 2012). Adult head muscle stem cells express *Pax7* (Harel et al., 2009; Sambasivan et al., 2009, reviewed in Sambasivan et al., 2011), and hence we used the emergence of *Pax7* expression in the head mesoderm as a sign that head muscle stem cells are being laid down. We first analyzed the onset of *Pax7* expression in the chicken head mesoderm, because chicken embryos are large and easy to obtain, and craniofacial muscle formation is well characterized in this model (reviewed in Noden and Francis-West, 2006). Using whole mount *in situ* hybridisation, we performed a time course for the expression of *Pax7* mRNA from the stage the head mesoderm is being laid down by the primitive streak at HH4 to stage HH24 when craniofacial muscle anlagen are well established (Noden et al., 1999; Camp et al., 2012; Figures 1A–L). Moreover, we analyzed the onset of Pax7 protein expression (Figure 1M and not shown) and we confirmed the association of *Pax7* expression domains with craniofacial skeletal muscle on serial frontal and cross sections (Figure 2).

We found that during early stages of development, *Pax7* expression was associated with the epiblast bordering the primitive streak, the neural plate border/ dorsal neural tube and emerging neural crest cells (Figures 1A–C). Expression in the dorsal neural tube remained high at later stages of development while expression in neural crest cells only persisted in the trigeminal ganglion and the frontonasal neural crest (Figures 1D–M, cns, V, ncc). In the trunk, from HH7 onwards *Pax7* was also expressed in the epithelialising somites (Figures 1B,C, arrowhead, and not shown), subsequently becoming confined to the muscle precursor/muscle stem cell lineage (Figures 1C–L). From HH16 onwards, *Pax7* was also expressed in migratory muscle precursors that leave the somites to form the hypoglossal/hypopharyngeal (Figures 1E–L, hg/hb) and limb musculature (not shown). From HH20 onwards, expression was found in the embryonic muscle stem cells that populate the myotome, drive both fetal and perinatal muscle growth and give rise to the trunk adult muscle stem cells (Gros et al., 2005; Relaix et al., 2005; Ahmed et al., 2006; Schienda et al., 2006) and not shown. These findings are in agreement with published data and underline the robustness of our approach.

Expression associated with craniofacial muscles was first seen at HH18 in the developing lateral rectus eye muscle (Figure 1F, lr), located just beneath the also *Pax7* positive trigeminal ganglion (Figures 1G–J, V, in G red staining for *Isl1*, Figure 2B, V). However, the lateral rectus is somewhat unusual as it is the only craniofacial muscle to express trunk markers such as *Paraxis* and *Lbx1* (Mootoosamy and Dietrich, 2002, and see below). In the anlagen of the other craniofacial muscles, *Pax7* staining did not emerge before day 3.5 of development. Expression was first seen in the ventral oblique eye muscle (Figures 1I,J, 2, vo), in strongly stained specimen followed by the anlagen of the jaw closure muscles (1^st^ pharyngeal arch = mandibular arch muscles; Figures 1J, 2, ma). Pax7 mRNA expression became more robust at E3.75/HH23 and E4/HH24 and eventually began to encompass all muscle anlagen, with the staining in the ventral and medial rectus lagging behind that of the other eye muscle anlagen (Figures 1K,L). However, signals were always weak compared to the expression in the frontonasal neural crest cells, the central nervous system and the trunk musculature. Moreover, expression of Pax7 protein in craniofacial muscle anlagen was delayed compared to the expression of *Pax7* mRNA and could only be detected from HH25 onwards (Figure 1M and not shown).

#### Comparison of Pax7 expression with the expression of markers for the early head mesoderm, for myogenic commitment and for myogenic differentiation

**Figure 3:**
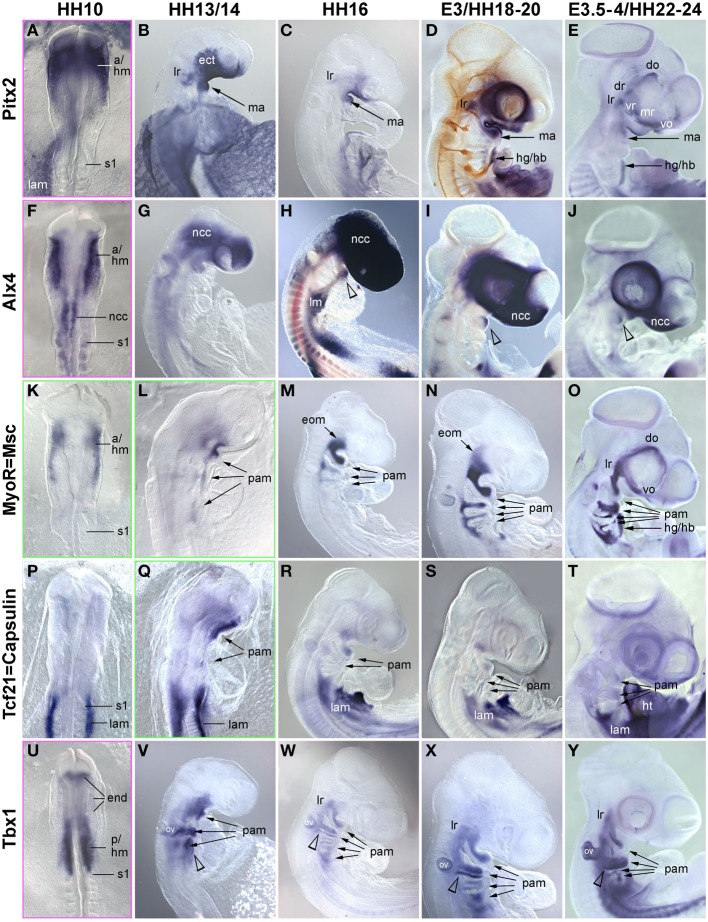
**Time course for the mRNA expression of head mesoderm markers in chicken embryos at HH10 (dorsal views) and HH13/14-E4 (lateral views); in (D), cranial nerves are revealed with the RMO270 antibody (brown staining)**. Gene names are displayed on the left of the panel; developmental stages are indicated at the top. The onset of marker gene expression is demarcated by a green frame, for genes being expressed earlier than HH10, frames are displayed in magenta. Abbreviations as in Figure 1 and: a/hm, anterior head mesoderm; ect, surface ectoderm; eom, extraocular muscle anlagen; end, endoderm; lam, lateral mesoderm; ht, heart; ov, otic vesicle; pam, pharyngeal arch muscle anlagen; p/hm, posterior head mesoderm. The open arrowhead in **(H,J)** points at *Alx4* expression in the mandibular arch ectoderm and in **(V–Y)** at *Tbx1* expression in the posterior ectoderm of the hyoid (2nd pharyngeal) arch. Note that all head mesoderm markers begin their expression well before *Pax7*. With the exception of *Alx4* which from HH13/14 onwards mainly labels cranial neural crest cells and *Tcf21/Capsulin* which throughout has lower expression levels than its paralog *MSc/MyoR*, all head mesoderm markers continue to strongly label the myogenic head mesoderm. Their expression domains are wider than that of *Pax7*, whose expression domain is nested in the expression domain of the head mesoderm genes (compare Figures 1, 4).

**Figure 4:**
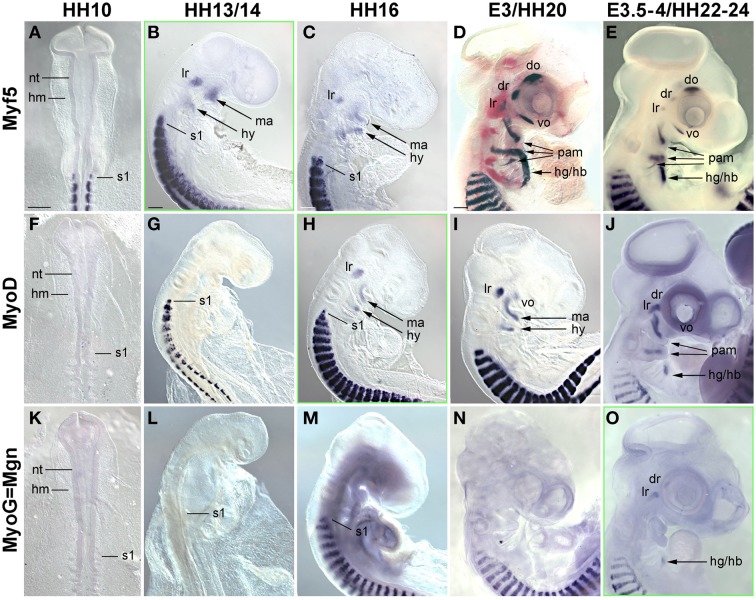
**Time course for the mRNA expression of Mrf transcriptions factors in chicken embryos from HH10-E4; orientation of specimen and frames indicating the onset of expression as in Figure 3**. Gene names and developmental stages are displayed as in Figure 3, Abbreviations as in Figures 1, 3 and: hy, hyoid arch. *Myf5* and *MyoD* indicate the myogenic commitment of precursor cells and are expressed in developing craniofacial muscle anlagen from HH13/14 (*Myf5*) and HH16 (*MyoD*) onwards, i.e., significantly before the onset of *Pax7*. Expression of *Myogenin* (*MyoG/Mgn*) indicates the entry of cells into muscle differentiation and commences at E3.5-4, i.e., about the same time as *Pax7* (compare with Figure 1).

**Figure 5:**
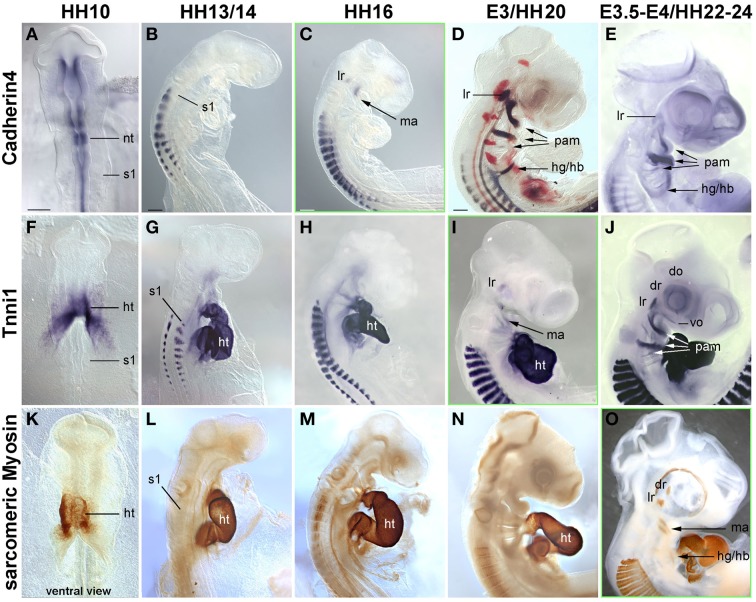
**Time course in chicken embryos from HH10-E4 for markers indicating the cohesion of muscle anlagen (Cadherin 4–mRNA expression) and terminal differentiation [Troponin I 1 (Tnni1)–mRNA expression; sarcomeric Myosin–MF20 antibody staining]**. Orientation of specimen and frames indicating the onset of expression as in Figures 3, 4; abbreviations as in Figures 1, 3, 4. The time course of *Cadherin* 4 expression resembles that of *MyoD*, and *Tnni1* expression commences in many craniofacial muscle anlagen E3, i.e., both are expressed before or at the onset of *Pax7* expression. Sarcomeric Myosins can be detected at E3.5-4, simultaneous to the onset of *Pax7*.

In the trunk, *Pax7* expression precedes the expression of any marker for myogenic commitment and differentiation (Jostes et al., 1991). However, the late onset of *Pax7* expression in the head musculature suggested that here, the sequence of marker gene expression and the set up of gene regulatory networks might be quite different. To explore this, we systematically analyzed the spatiotemporal distribution of early head mesoderm markers (*Pitx2*, *Alx4*, *M*usculin = Msc = MyoR, Tcf21 = Capsulin, *Tbx1*; Figure 3), of markers indicating the onset of myogenesis (*Mrf* family members; Figure 4), and of markers indicating cohesion and terminal differentiation of muscle anlagen (*C*adherin4 = Cdh4 = R−Cadherin, *Tnni1*, sarcomeric Myosin; Figure 5).

##### Markers for the cranial mesoderm

The early chicken head mesoderm is known to expresses the transcription factors Pitx2, Alx4, Msc, Capsulin, and Tbx1 (Bothe and Dietrich, 2006; von Scheven et al., 2006; Bothe et al., 2011), and in accord with this work, mRNA expression of *Pitx2* and *Tbx1* was first seen at HH6 in distinct rostro-caudal regions of the head mesoderm (not shown). At HH9 *Alx4* expression emerged within the confines of the *Pitx2* territory, followed by *Msc* expression at HH10. Between HH10 (Figures 3A,F,K,P,U) and HH13/14 (Figures 3B,G,L,Q,V), *Tbx1* expression spread anteriorly and *Msc* expression spread posteriorly, co-labeling the branchiomeric mesoderm and the anlage of the caudal-most eye muscle, the lateral rectus. Also at HH13/14, *Tcf21* expression commenced in the anlagen of the branchiomeric muscles, but remained weaker than that of its paralog *Msc* throughout (Figure 3Q). *Alx4* on the other hand became strongly upregulated in craniofacial neural crest cells, thus, masking any residual mesodermal expression (Figures 3G–J, ncc). Owing to these changes, *Pitx2*, *Msc*, and *Tbx1* became the most prominent markers for the myogenic head mesoderm, between HH16 to HH22-24 labeling the anlagen of the eye and mandibular arch muscles (*Pitx2*, Figures 3C–E), all eye and branchiomeric muscles (*Msc*; Figures 3M–O) or the branchiomeric muscles and the lateral rectus eye muscle (*Tbx1*, Figures 5W–Y). Respective expression domains for these markers were wider than those for *Pax7* but included the *Pax7* domains.

##### Markers for myogenic commitment and the initiation of myogenesis

In the developing chicken somites, commitment to myogenesis and entry into differentiation is demarcated by the sequential expression of the *MyoD* family of *Mrf* genes, with *Myf5* commencing first, followed by *MyoD*, *MyoG* and *Mrf4* (Berti and Dietrich, unpublished observations). In the avian head mesoderm, expression of the *Mrf* family commenced at HH13/14, when *Myf5* labeled the anlagen of the lateral rectus and the mandibular and hyoid arch muscles (Figure 4B, see also Noden et al., 1999). At HH16, expression of *Myf5* was accompanied by that of *MyoD* (Figures 4C,H). Between HH20-HH24, all craniofacial muscle anlagen began to express these genes, with *MyoD* expression always following that of *Myf5* (Figures 4D,E,I,J). *MyoG* expression was detected at HH22-24 (Figure 4O), yet *Mrf4* was still silent at this stage (not shown). Thus, for all craniofacial muscles, including the peculiar lateral rectus eye muscle, expression of *Myf* and *MyoD* emerged well before that of *Pax7*; and *MyoG* expression began at approximately the same time as *Pax7*. This is different from the trunk where *Pax7* is expressed before any of the *Mrfs*.

##### Markers for muscle cohesion and terminal differentiation

Cadherin 4 has been shown to act in the communication and differentiation of myogenic cells (Rosenberg et al., 1997) and to be expressed in the chicken lateral rectus eye muscle (Mootoosamy and Dietrich, 2002). Troponins and sarcomeric Myosins are components of the contractile proteins complexes in both cardiac and skeletal muscle, with Tnni1 specifically acting in the early developing slow-twitch muscle and (during embryogenesis) in the heart (see http://geisha.arizona.edu/geisha/). We therefore used these markers as indicators for the cohesion and terminal differentiation of muscle anlagen. Expression of these markers was first seen at HH16, when *Cadherin4* labeled the lateral rectus and mandibular arch muscle anlagen (Figure 5C). *Tnni1* expression commenced at HH20, at HH22-24 encompassing all craniofacial muscle anlagen (Figures 5I,J). At this stage, sarcomeric Myosins were also expressed, indicating the presence of functional skeletal muscle (Figure 5O and Noden et al., 1999). The onset of *Cdh4* and *Tnni1* expression before or concomitant with that of *Pax7* suggests that in the head, the process of skeletal muscle development is well under way when the *Pax7* cell lineage is being established.

#### Comparison of Pax7 expression with the expression of trunk premyogenic genes

**Figure 6:**
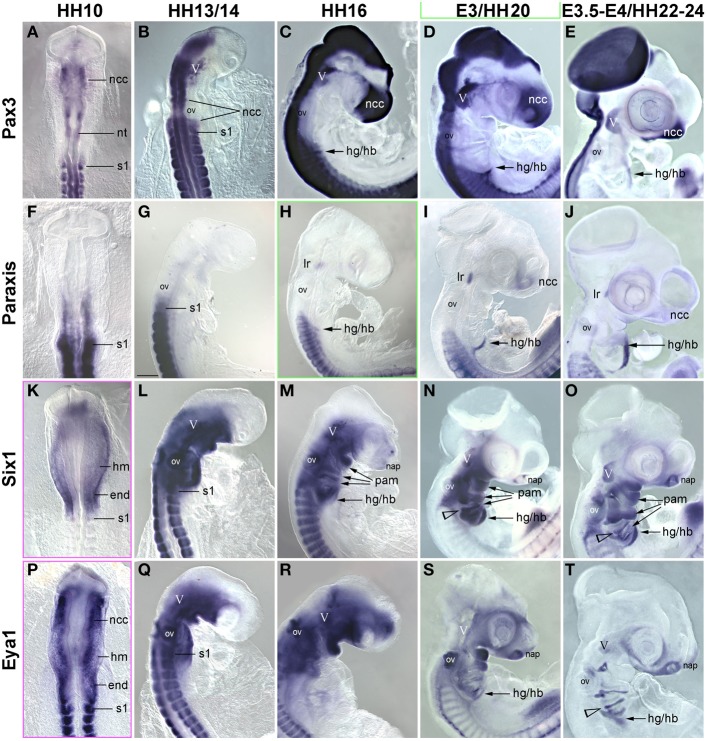
**Time course for the mRNA expression of trunk pre-myogenic genes; embryos are displayed and annotated as in Figures 3–5**. Abbreviations as before and: na, nasal pit. **(A–E)** *Pax3* labels the central nervous system, the frontonasal neural crest, the trigeminal ganglion, the somites and the somite-derived hypobranchial and limb muscle precursors, but remains absent from genuine craniofacial muscle anlagen. **(F–J)** *Paraxis* expression overlaps with that of *Pax3* and *7* in the somite-derived muscle precursors and in the frontonasal crest. Similar to *Pax7*, *Paraxis* is also expressed in the lateral rectus eye muscle, but is absent from all other craniofacial muscles. *Six1* **(K–O)** and *Eya1* **(P–T)** are expressed in the head mesoderm before and at HH10. From that stage onwards mesoderm expression becomes somewhat obscured by the overlying expression in neural crest cells. However, *Six1* (but not *Eya1*) remains detectable in craniofacial muscle anlagen.

In previous studies, we had shown that the early head mesoderm does not express the *Pax7* paralog *Pax3* (Mootoosamy and Dietrich, 2002; Bothe and Dietrich, 2006). However, stages at the onset of *Pax7* expression have not been analyzed. Moreover, in the trunk Paraxis, Six1 and the Six1 co-activator Eya1 also act as premyogenic regulators (Wilson-Rawls et al., 1999; Grifone et al., 2005, 2007; Relaix et al., 2013). To explore if any of these genes might be in the position to serve as intermediaries between the head mesoderm genes, the *Mrf* genes and *Pax7*, we investigated the expression of these trunk premyogenic genes (Figure 6). Our analysis revealed that *Pax3* expression overlapped with that of *Pax7* in the neural tube, the trigeminal ganglion, the frontonasal neural crest and somites, but remained absent from craniofacial skeletal muscle anlagen (Figures 6A–E). *Paraxis* expression overlapped with the expression of *Pax3* and *Pax7* in the frontonasal neural crest cells and the somites, but, with the exception of the lateral rectus muscle, was also not expressed in craniofacial muscle anlagen (Mootoosamy and Dietrich, 2002; Figures 6F–J). *Six1* showed a widespread expression, at HH5-10 encompassing the preplacodal ectoderm, both the mesoderm and the endoderm underneath the neural plate, and weakly, the developing somites (shown for HH10, Figure 6K). From HH13/14 onwards (Figures 6L–O), expression was strong in the otic vesicle and nasal pit, the trigeminal placodes, the posterior edge of the 2–4th pharyngeal arches and the pharyngeal pouches, the somites and the emerging migratory muscle precursors. Moreover, low-level, widespread *Six1* expression was found throughout the head mesenchyme. However, expression was also found in the anlagen of branchiomeric muscles, with strongest expression in the hyoid arch. Expression of *Eya1* (Figures 6P–T) was similar to that of *Six1*. Yet, while strong and lasting expression was detected in craniofacial neural crest cells, expression levels in craniofacial muscle anlagen declined. This suggests that in contrast to the other trunk pre-myogenic genes, *Six1* and *Eya1* may play an–albeit more subordinate than in trunk–role in head skeletal muscle development and may influence muscle development indirectly via the control of connective tissue development. Yet none of the trunk pre-myogenic markers seems to take over from the head mesoderm genes to prepare for myogenesis and/or muscle stem cell deployment.

### Emergence of Pax7 expressing myogenic cells in mouse craniofacial muscles

#### Time course of murine Pax7 expression

**Figure 7:**
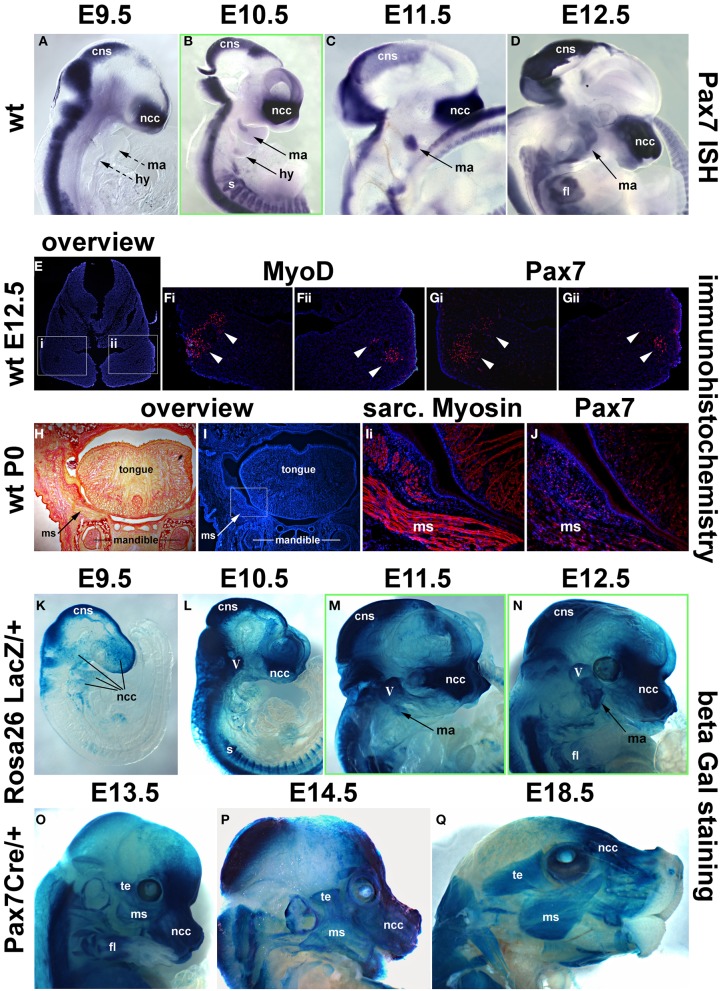
**Time course of *Pax7* expression in the mouse. (A–D)** *Pax7* mRNA expression from E9.5-E12.5 of development; lateral views of the right side of embryos, anterior to the top. Expression is readily detectable in the developing central nervous system, emigrating neural crest cells (prolonged expression in the frontonasal neural crest) and the somites. Head muscle anlagen show expression first at E10.5. **(E)** Serial cross sections of the mandibular arch at E12.5, dorsal to the top; **(F,G)** higher magnifications of the areas indicated by the boxes in **(E)** and stained for Dapi and MyoD protein **(Fi,ii)** or Dapi and Pax7 protein **(Gi,ii)**. Note that MyoD and Pax7 domains overlap. **(H,I)** Serial frontal sections of the mandible at birth (P0), dorsal to the top, lateral to the left. **(H)** Sirius Red staining showing muscle fibers in yellow and bone and connective tissue in red. **(I)** Dapi staining of the same region, with **(Ii)** showing a magnification of the cheek and the floor of the mouth as indicated in **(I)**. Skeletal muscle fibers are shown in red. **(J)** Subsequent section stained for Pax7 protein in red. Note the punctate, nuclear staining for Pax7, associated with the Myosin-positive muscle fibers. **(K–Q)** Lineage tracing of *Pax7* expressing cells, revealed by beta galactosidase staining; lateral views of the right side of embryos, dorsal to the top. With a delay of 1 day, cells with a history of *Pax7* expression can be detected in the central nervous system, the trigeminal ganglion, the frontonasal neural crest and the somites. In craniofacial muscle anlagen, cells with a history of *Pax7* expression can be detected between E11.5 and E12.5, with a more robust staining appearing at E13.5. Eventually, all craniofacial muscles are stained and the staining is found in muscle fibers, indicating that, similar to the trunk, *Pax7*-positive cells contribute to fetal and perinatal muscle growth. Abbreviations as in Figures 1, 3, 4 and: fl, forelimb; ISH, *in situ* hybridisation; ms, masseter; te, temporalis muscle; wt, wildtype.

Our analysis in the chicken suggested that, in contrast to the trunk, *Pax7* expressing cells associated with cranial skeletal muscle emerge late, well after the onset of markers for myogenic commitment and at the time that cells begin to enter terminal differentiation. To explore whether this unexpected timing is true also for other amniotes, we next investigated the mouse, establishing both the onset of mRNA (Figures 7A–D) and protein expression (Figures 7E–G; see also Figure 9). Moreover, we investigated the position of Pax7 protein expressing cells at birth (Figures 7H–J) and analyzed the fate of *Pax7* positive cells from embryonic to late fetal stages of development (Figures 7K–Q).

As in the chicken, mouse *Pax7* expression was seen in neural crest cells, the central nervous system and in somites from early neurulation stages onwards (Jostes et al., 1991, and not shown). At E9.5, even though the first two pharyngeal arches were well developed, there was no expression in their myogenic mesodermal core (Figure 7A, ma, hy; dotted arrows). Expression in the pharyngeal arch muscle anlagen was first seen at E10.5, and became more robust between E11.5 -12.5 (Figures 7B–D, ma, hy; solid arrows). Also similar to the chicken, expression of Pax7 protein was delayed compared to the expression of mRNA and was first detected at E12.5, with the Pax7 domain overlapping with that of MyoD (Figures 7E–G). Both at E12.5 and in the newborn, Pax7 protein was located in the nuclei of cells (note the punctate staining in Figures 7Gi,Gii,J). In the newborn, the Pax7 staining was associated with muscle fibers revealed by antibodies detecting sarcomeric Myosins (compare Figures 7Ii,J), in line with studies that showed that at this stage, *Pax7* expressing cells had assumed their mature satellite cell (adult muscle stem cell) position (Harel et al., 2009; Sambasivan et al., 2009).

Using the *Pax7 Cre* driver and the *Rosa26R^LacZ^* reporter, we traced cells that in their past expressed robust levels of *Pax7* (Hutcheson et al., 2009). This approach allowed to visualize the contribution of *Pax7* expressing neural crest cells to the trigeminal ganglion, the pharyngeal arches and the developing frontonasal skeleton with a delay of 1 day compared to the onset of mRNA expression; likewise, the *Pax7* cell lineage in the central nervous system and in the somites could readily be traced with a delay of 1 day (Figures 7K–L, and not shown). LacZ positive cells contributing to the mandibular arch muscle anlagen were just about detectable at E11.5 (Figure 7M, ma, arrow), at E12.5, this contribution was more evident (Figure 7N, ma, arrow). By E13.5, virtually all developing craniofacial muscles had received a contribution of cells that once had expressed *Pax7* (compare Figure 7O and Figure 9D, and see also Figures 9F,G,I), and at E14.5 (Figure 9P) and E18.5 (Figure 9Q), the cells had contributed to muscle fibers. This suggests that at fetal and perinatal stages of development *Pax7* positive cells contribute to the growth of head skeletal muscle in a similar fashion as in the trunk, inferring a convergence of developmental pathways.

#### Comparison of Pax7 expression with the expression of head mesoderm and myogenic markers

**Figure 8:**
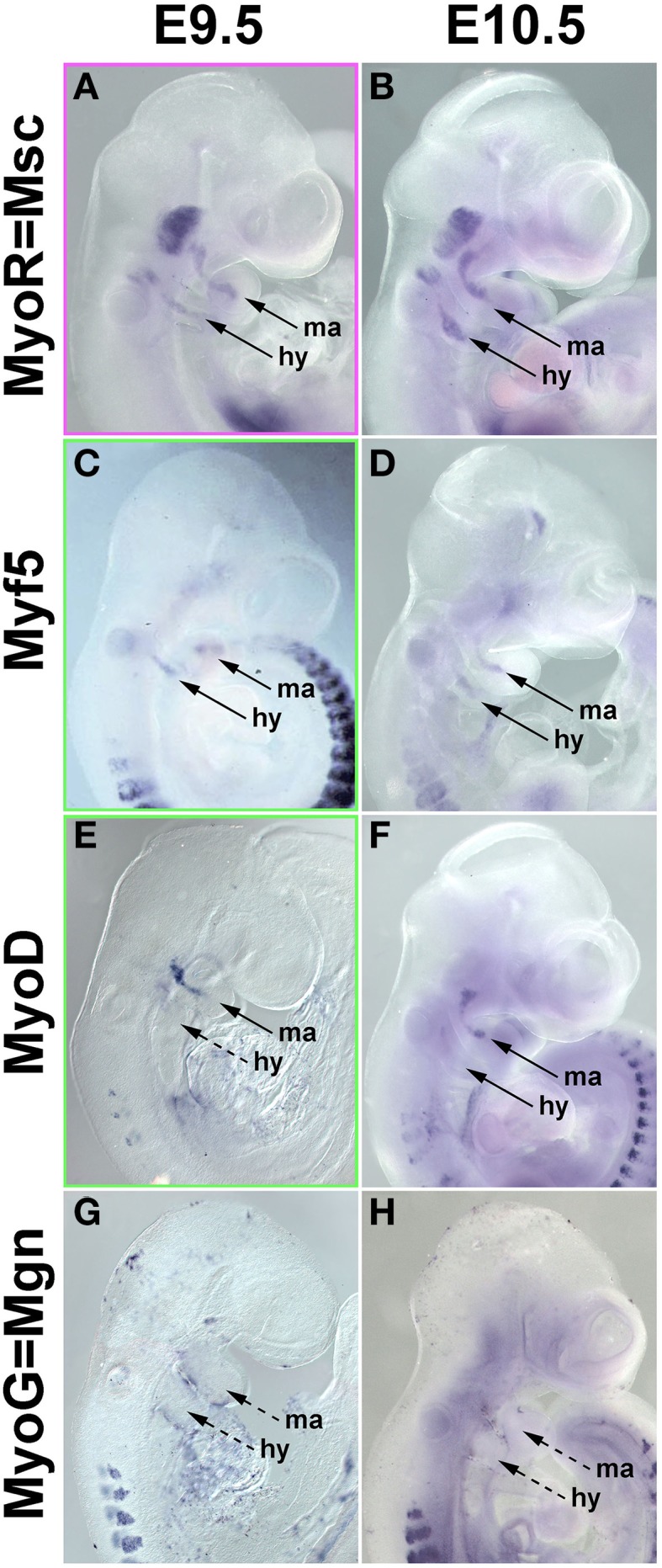
**mRNA Expression of mouse head mesoderm markers and markers for myogenic commitment before and at the onset of *Pax7* expression**. Lateral views, dorsal to the top. Stages of development are indicated at the top of the panel, gene names on the left. **(A,B)** *Musculin* expression commences before E9.5 (not shown); at E9.5-10.5, the gene is widely expressed in the myogenic head mesoderm. *Myf5* **(C,D)** and *MyoD* **(E,F)** expression commences at E9.5, i.e., before the onset of *Pax7*. **(G,H)** *Myogenin* expression is not yet detectable at these stages and commences slightly later at E11.5 (not shown).

In the chicken, *Pax7* expression in craniofacial muscle anlagen commenced after the onset of head mesoderm markers and after the onset of *Myf5* and *MyoD*. To explore whether this is also true for mammals, we investigated the expression of *Pitx2*, *Tbx1*, *Msc*, *Myf5*, *MyoD*, and *MyoG* at E7.5-E10.5 of development (Figure 8 and not shown). Expression of *Pitx2*, *Tbx1*, and *Msc* commenced much earlier at E7.5-8 (not shown). At E9.5, *Msc* labeled the myogenic cells that will engage with the eye as well as the core of the first two pharyngeal arches; the same pattern was found at E10.5 (Figures 8A,B). Significantly, at both stages, both *Myf5* as well as *MyoD* were well expressed in the anlagen of first and second arch muscles (Figures 8C–F), while *MyoG* was not yet active (Figures 8G,H). Thus, in both amniote models, the expression of the head mesoderm markers preceded the expression of *Myf5* and *MyoD*, which in turn preceded the expression of *Pax7*.

#### Pax7 expression in regions with a history of MyoD expression

**Figure 9:**
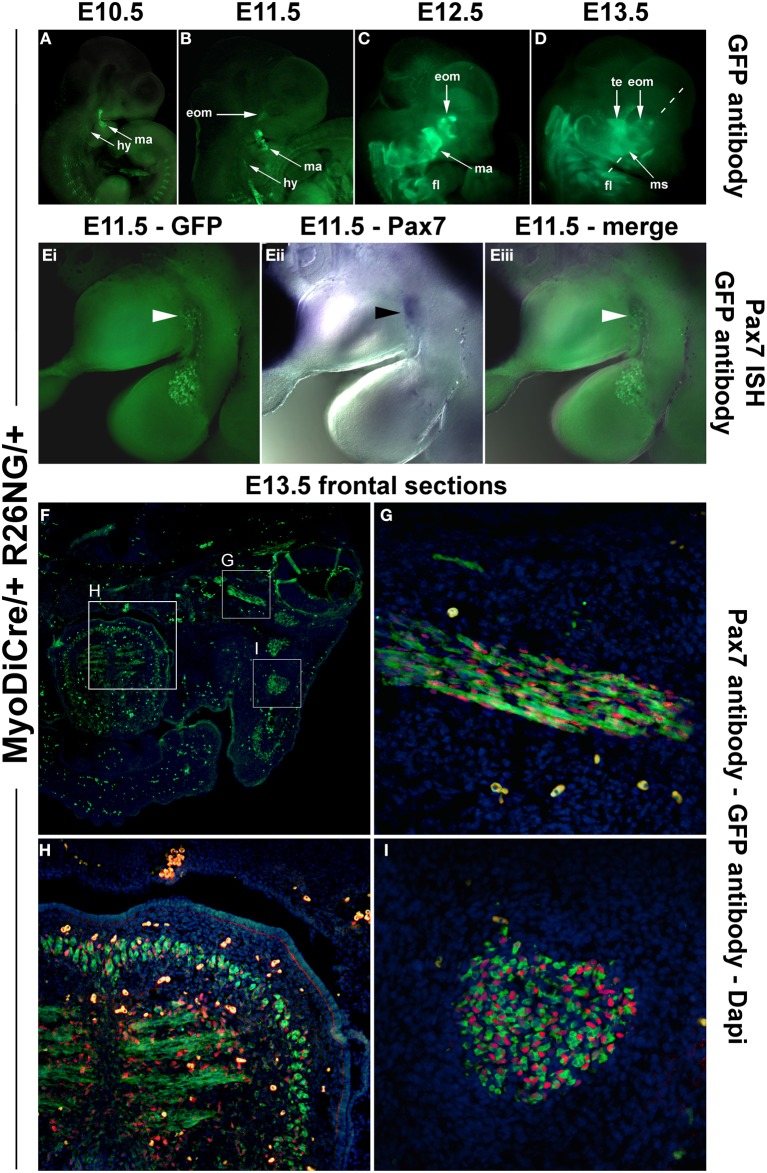
**Lineage tracing of *MyoD* expressing cells in MyoDiCre/+ R26NG embryos, revealed by anti-GFP antibody (green) staining. (A–D)** Lateral views of the right side of E10.5-E13.5 embryos; the dotted line indicates the sectional plane in **(F–I)**. **(Ei-iii)** Lateral views of the left side of an E11.5 embryo, stained for *Pax7* mRNA (blue) and GFP protein (green); dorsal to the top. **(F)** Frontal section of an E13.5 embryo, stained for Pax7 protein (red), GFP (green), and Dapi (blue). **(G)** Detail of the ventral rectus eye muscle, **(H)** detail of the tongue, **(I)** detail of the masseter as indicated in **(D,F)**. The widely distributed bright green **(F)** or yellow cells **(G–I)** are autofluorescing blood cells. Cells with a history of *MyoD* expression can readily be detected at E10.5 and 11.5, first in the mandibular and hyoid arch, then in the developing extraocular muscles. In head-mesoderm-derived muscles, *Pax7* mRNA and subsequent protein expression colocalises with that of MyoD-Cre driven GFP, and Pax7 containing nuclei reside in GFP expressing cells. In contrast, in the somite-derived tongue muscle, most Pax7-positive nuclei are not located in GFP expressing cells. Abbreviations as in Figures 1, 4, 5 and: eom, developing extraocular muscles.

Since *MyoD* expression in craniofacial muscle anlagen preceded the onset of *Pax7*, we began to explore whether *MyoD* might be upstream of *Pax7*, similar to what has been shown for P19 EC cells misexpressing *MyoD* (Gianakopoulos et al., 2011). For this we turned to the *MyoDiCre* mouse driver line (Kanisicak et al., 2009; Wood et al., 2013). We first established when the *Rosa26 GFP* reporter (R26NG; (Yamamoto et al., 2009) may reveal activity of the MyoDiCre driver in craniofacial muscle anlagen; we found that this was the case from E10.5 onwards (Figures 9A–D). At E12.5 and E13.5, the GFP expression pattern was highly similar to that of *Pax7* mRNA and *Pax7*-driven LacZ (compare Figures 9C,D, 7D,N,O). To test whether the *Pax7* mRNA we had detected earlier at E11.5 might colocalise with the GFP read-out of the *MyoD* locus, we simultaneously visualized the *Pax7* mRNA and GFP driven by MyoDiCre (Figures 9Ei–iii). We found that indeed, cells with a history of *MyoD* expression engulfed the *Pax7* domain located at the maxillary-mandibular junction. To directly test whether *Pax7* expressing cells have a history of *MyoD* expression, we stained for Pax7 and GFP proteins on cryosections of MyoDiCre/+; R26NG/+ embryos at E12.5 (not shown) and E13.5 (Figures 9F–I). This revealed that not all cells with a history of *MyoD* expression also expressed Pax7. However, for head mesoderm-derived muscles, the majority of Pax7-positive nuclei were located in cells with MyoDiCre driven GFP expression (Figures 9G–I). In contrast, in the somite-derived tongue muscle most Pax7-positive nuclei were in GFP-negative cells (Figure 9H). Taken together, our data support the idea that head mesodermal cells express early *Mrf* and become myogenic before turning on *Pax7*.

### Emergence of Pax7 expressing myogenic cells in anamniote craniofacial muscles

Our analysis suggested that in amniotes, cells that eventually will populate the head muscle stem cell niche are being deployed after, possibly from, cells committed to skeletal muscle formation. However, in amniotes, overall skeletal myogenesis is delayed compared to anamniotes that rely on functional muscle during larval stages of development. Therefore, we investigate the emergence of *Pax7* positive cells in craniofacial muscles of two anamniote models, the African clawed frog *X. laevis* (a sarcopterygian vertebrate like mouse and chicken) and the teleost fish *D. rerio* (zebrafish, an actinopterygian vertebrate).

#### Emergence of Pax7 expressing myogenic cells in craniofacial muscles of xenopus laevis

**Figure 10:**
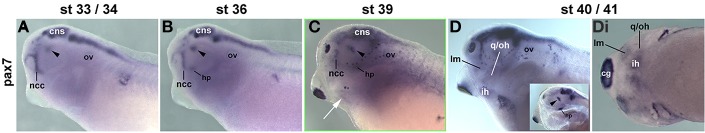
**Time course of *pax7* mRNA expression in *Xenopus laevis***. Lateral views, anterior to the left. Embryonic stages are indicated at the top. Inset in **(D)**: pharyngeal arches and head mesenchyme were dissected away from the left side to reveal the brain. Up to stage 36, Pax7 expression is confined to the central nervous system including the ventral diencephalon (arrowhead), the hypophysis (hp), and the frontonasal neural crest cells. Weak expression is also seen in the somites. From stage 39 onwards, weak expression can be detected in craniofacial muscle anlagen. Abbreviations as before and: cg, cement gland; hp, hypophysis; ht, heart; first arch derived muscle: im, m. intermandibularis anlage; lm, m. levatores mandibulae anlage; second arch derived muscle: ih, m. interhyoideus anlage; oh, m. orbitohyoideus anlage; qh, m. quadrato-hyoangularis anlage; q/oh, common oh and qh precursor.

In *X. laevis*, the genome was duplicated upon hybridisation between two ancestral species approximately 65 million years ago, and extant *X. laevis* is considered allotetraploid (Hughes and Hughes, 1993; Evans et al., 2004; reviewed in Evans, 2008). However, when we cloned partial cDNA sequences of the two duplicate *pax7* genes, they had an identity of 87% (data not shown). Moreover, the *pax7a* probe had an identity of 85.5% and the *pax7b* probe of 78.7% with the corresponding sequence of the single *Xenopus tropicalis pax7* gene, this however only shared 55% of nucleotides with its paralog *pax3*. Correspondingly, *in situ* hybridisation of *X. laevis* embryos with the *pax7a* and *b* probes alone, with a mix of both probes or with the *X. tropicalis pax7* probe produced the same expression patterns, and hence only the data for the *X. tropicalis* probe are being shown (Figure 10). This analysis revealed expression in the central nervous system, in craniofacial neural crest cells, in the ventral diencephalon and the hypophysis as well as in the somitic dermomyotome, recapitulating the data by Maczkowiak et al. (2010), Daughters et al. (2011), Della Gaspera et al. (2012), Bandin et al. (2013) (Figures 10A,B and data not shown). *Pax7* expression in areas of developing head muscle anlagen was detected from stage 39 onwards and became somewhat stronger at stages 40/41 [Figures 10C–Di; muscles were identified according to Ziermann and Olsson (2007) and Schmidt et al. (2013)]. However, expression levels remained low compared to other expression domains.

**Figure 11:**
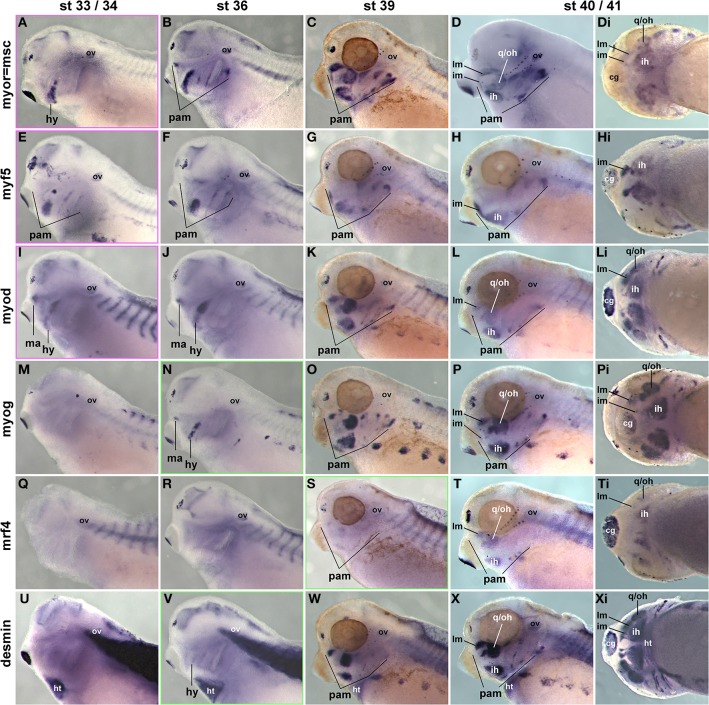
**Time course of head mesoderm and muscle gene expression in *Xenopus laevis***. Same stages, views, and abbreviations as in Figure 11; markers are indicated on the left. Note that *msc*, *myf5*, *myod*, *myog*, and *desmin* are expressed before, *mrf4* concomitant with the onset of *pax7* expression.

Previous studies have investigated the expression of some mesodermal and myogenic markers in the *Xenopus* embryos (Della Gaspera et al., 2012), but a systematic comparison with *pax7* has not been carried out. We therefore cloned probes for the head mesodermal gene *msc*, for all *mrf* genes and for the muscle structural gene *desmin*. As expected, *msc* labeled the myogenic head mesoderm from early stages onwards (Figures 11A–Di and not shown); the exception is the mesoderm of the first arch which however is *pitx2* and *tbx1* positive (Della Gaspera et al., 2012). These markers are followed by the expression of *myf5* and *myod* (Figures 11E–Hi,I–Li and not shown). *myf5* and *myod* showed overlapping but non-identical expression patterns, with *myf5* strongly labeling the 1st arch derived intermandibularis muscle anlage (Figures 11H,Hi, im) and *myod* the 1st arch derived levator mandibularis muscle (Figures 11L,Li, lm). Yet when *myog* and *desmin* expression commenced in developing head muscles at st36, the markers encompassed all craniofacial muscle anlagen, and they were followed by *mrf4* expression at st39 (Figures 11M-Pi,Q-Ti,U-Xi). Thus, as in the two amniote models, frog *pax7* expression in craniofacial muscle anlagen began late, after the commitment of cells to myogenesis and the onset of differentiation.

**Figure 12:**
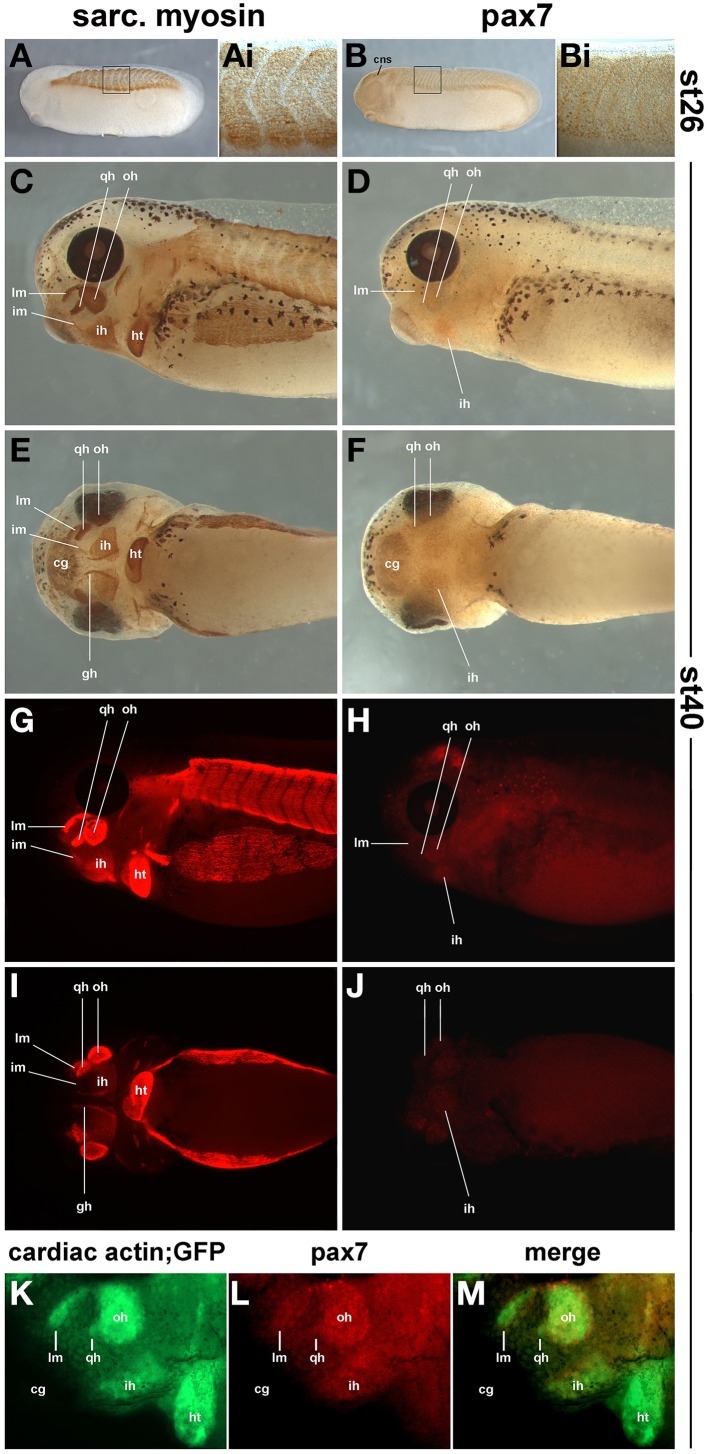
**Sarcomeric myosin (A,C,E,G,I) and pax7 protein expression (B,D,F,H,J) in st26 *Xenopus laevis* control embryos and in craniofacial muscle anlagen embryos at st40. (K–M)** GFP expression and pax7 protein expression in a st40 cardiac actin; GFP embryo. **(A,B,C,D,G,H,K,L)** lateral views, rostral to the left, dorsal; to the top; **(E,F,I,J)** ventral views, rostral to the left. In **(A–F)** a HRP-coupled secondary antibody was used, in **(G–J)** a secondary antibody coupled to Alexa fluor 594. In **(K–M)**, Alexa fluor 488 and 594 coupled secondary antibodies were used to detect anti GFP and pax7 primaries, respectively. The staining at st26 recapitulates the known myosin and pax7 expression patterns. At st40, the strong expression of sarcomeric myosin and the activity of the cardiac actin promoter indicate that in particular in muscle anlagen associated with the first (mandibular) and second (hyoid) pharyngeal arch, muscle differentiation is well under way. In these muscle anlagen, a faint expression is visible for pax7. The pattern is punctuate, as expected for a nuclear localisation of the pax7 protein. Abbreviations as in Figures 1, 10, 11 and: somite derived muscle: gh, m. geniohyoideus anlage.

In order to determine the onset of *pax7* protein expression and to ascertain that expression domains are associated with skeletal muscle, we compared the expression of sarcomeric myosins (MF20 antibody staining, Figures 12A,C,E,G,I) and the read-out of the cardiac actin promoter (cardiac actin; GFP frogs; Figure 12K) with that of pax7 protein (Figures 12B,D,F,H,J,L,M). To associate expression with anatomical features, a diaminobenzidine staining was performed (Figures 12A–F); to better detect signals away from the surface, a fluorescent secondary antibody was used (Figures 12G–J,L,M). As a control, we performed an antibody staining at st26, focusing on the somitic expression (Figures 12A,Ai,B,Bi); this recapitulated the data by Daughters et al. (2011). Our stainings at st40 revealed pax7 protein expression in craniofacial muscle anlagen, with expression levels being significantly lower than those of sarcomeric myosins (Figures 12C–J).

#### Emergence of Pax7 expressing myogenic cells in craniofacial muscles of the zebrafish

Chicken, mouse and frog all belong to the lobe-finned/limbed (sarcopterygian) class of osteichthyans, while zebrafish is a teleost that belongs to the ray-finned (actinopterygian) class (Clack, 2002). Thus, zebrafish is the model most distantly related to humans/ mammals. Teleosts have undertaken a 3rd genome duplication 350 million years ago (Postlethwait, 2007), and retained both *pax7* copies (Seo et al., 1998). The coding sequences of these genes are 80.9% identical, and they share 83.6 and 82.6% (*pax7b*) identity with coding sequence of the single *pax7* gene in the spotted gar, a holost fish (data not shown). Yet zebrafish *pax7a* sequences are 63.5/57.3% identical with *pax3a/b* sequences, and *pax7b* sequences are 61.3/56.2% identical with those from *pax3a/b*, respectively. This suggests that *pax7a* and *b* are likely to cross-hybridize with the mRNA of the duplicate gene but not with *pax3a/b* mRNAs. Here, we used a *pax7a* probe as it provides a more robust signal that the *pax7b* probe (Hughes, personal communication). This probe and the pax7 antibody (see below) had been used earlier (Seo et al., 1998; Hammond et al., 2007) and recapitulated *pax7* expression in the nervous system and somites as displayed in these studies (not shown).

**Figure 13:**
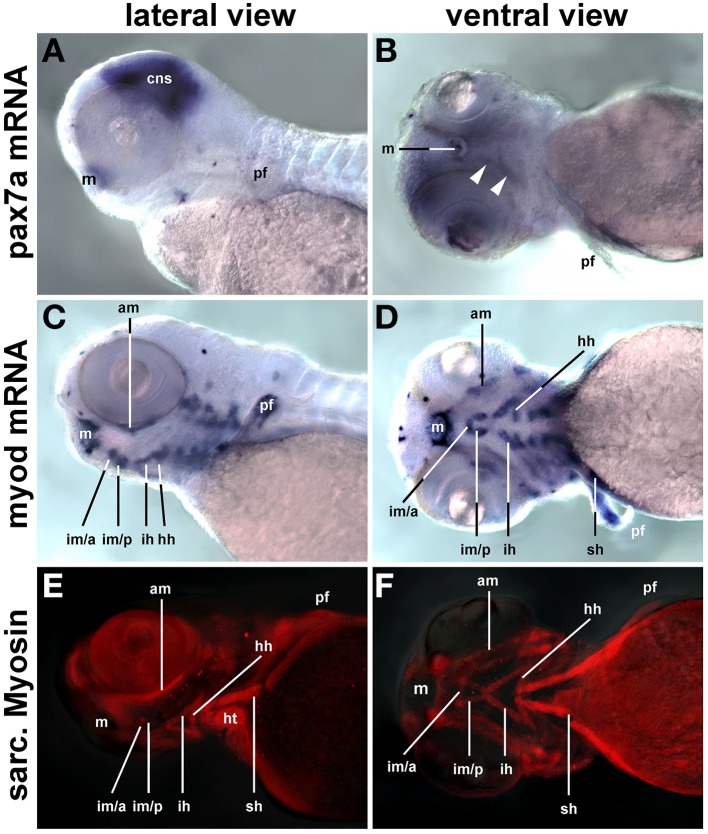
***pax7a* mRNA, *myod* mRNA, and sarcomeric myosin expression (MF20 antibody staining) in 72 hpf zebrafish larvae, lateral views, anterior to the left; markers are indicated on the left of the panel**. While myogenic markers show robust expression in craniofacial muscle anlagen, *pax7* mRNA is barely detectable (arrowheads). Abbreviations as before and: am, adductor mandibulae; hh, hyohyoideus; hpf, hours post fertilization; ih, interhyoideus; im/a, intermandibularis anterior muscle; im/p, intermandibularis posterior muscle; m, mouth; pf, pectoral fin; sh, sternohyoideus.

Craniofacial muscle anlagen begin to express *myf5* and *myod* at 24 and 32 h post fertilization (hpf), with *myod* showing a more widespread expression (Lin et al., 2006; Hinits et al., 2009) and data not shown). *myog* and *mrf4* are readily detectable at 48 hpf, and when at 72 hpf the animals rely on their head muscles to ventilate the gills and feed, structural proteins are well established (Schilling and Kimmel, 1997). In contrast, *pax7a* expression in craniofacial muscles was barely detectable at 72 hpf (Figures 13A,B, arrowheads) whereas expression of *myod* mRNA (single copy gene; Figures 13C,D) and of sarcomeric myosins (MF20 antibody staining, Figures 13E,F) was very strong at this stage. Thus, also in teleosts *pax7* expressing future head muscle stem cells arise late and possibly after the cells committed to myogenesis.

## Discussion

The vertebrate head mesoderm is a unique type of mesoderm as it forms both skeletal muscle and cardiac tissue. Specifically, the head mesoderm lining the lateral and ventral aspects of the pharynx retains the ability to contribute to skeletal muscle and the heart for a prolonged period of time, and it contributes the ventral muscles of the pharyngeal arches and the outflow tract of the heart (reviewed in Sambasivan et al., 2011). It is conversely debated whether in amniotes including humans, there are any proliferative cells in the mature heart; it is clear however that in contrast to for example the zebrafish (an anamniote) amniote heart regeneration is currently not possible (reviewed in Garbern and Lee, 2013). Yet, adult head skeletal muscle stem cells, besides expressing the muscle stem cell marker *Pax7*, retain the expression of the early head mesodermal markers (Harel et al., 2009; Sambasivan et al., 2009). These cells, when transplanted into trunk skeletal muscle lose their head-specific expression profile and contribute to trunk muscle regeneration. However, it is appealing to explore whether in the appropriate environment or niche, cells may be able to repair muscle in dystrophies predominantly affecting head muscles, or could be reprogrammed to regenerate the heart. Given that cardiovascular diseases are the predominant cause of death in the Western world (Garbern and Lee, 2013), the latter is of great medical importance.

Prerequisite to exploring the properties and therapeutic potential of head muscle stem cells is an understanding of their developmental biology. However, while some inroads into the unraveling of head skeletal muscle development have been made (reviewed in Sambasivan et al., 2011), timing and mechanisms controlling head muscle stem cell deployment are still largely unknown. Muscle stem cells rely on the expression and function of the *Pax7* gene, and *Pax7* is currently the most reliable marker for muscle stem cells (Seale et al., 2000; Kassar-Duchossoy et al., 2005; Relaix et al., 2006; Lepper et al., 2009; von Maltzahn et al., 2013). In this study, we used a comparative approach in the commonly used vertebrate models for myogenesis, chicken, mouse, *Xenopus* and zebrafish, and established, when and how *Pax7* expressing head muscle stem cells emerge. These models represent both the lobe-finned/ limbed (sarcopterygian) and ray-finned (actinopterygian) class of “bony” (osteichthyan) vertebrates, and any shared characteristics point at evolutionarily conserved, basic mechanisms.

### Head muscle stem cells arise late in the development

In vertebrates, the longitudinal body axis is laid down sequentially during gastrulation, proceeding from anterior to posterior (reviewed in Gilbert, 2000). The head therefore is always developmentally advanced, yet head skeletal muscles are known to develop late (Sambasivan et al., 2011). In tune with this delay, we found that *Pax7* expressing head muscle stem cells also develop late. However, this delay is not proportionate: the avian embryo, for example, takes 21 days to develop, the head mesoderm is being laid down within the first day, the final pattern of head mesoderm markers is established within a further day, yet it takes about 1.5 days to *Pax7* mRNA expression and another 12 h for readily detectable protein levels. This discrepancy is even more pronounced in anamniotes where *Pax7* levels are low throughout and first detectable around the time of larval hatching. This suggests that the head mesoderm undertakes a series of so far ill-defined steps before head muscle stem cells can be deployed.

### Head muscle stem cells arise after the onset of Myf5 and MyoD expression

Using the aid of marker genes, we investigated the processes the head mesoderm is engaged in before the onset of *Pax7*. Notably, in all models examined here, the myogenic head mesoderm expresses *Myf5* and/or *MyoD* (amniotes: co-expression, anamniotes: coexpression in most, but differential expression of *myf5* or *myod* in selected muscle anlagen) before *Pax7*, indicating that the majority of cells have committed to a skeletal muscle fate. Moreover, several markers indicating the onset of myogenic differentiation are also expressed before the onset of *Pax7*. This is particularly evident in anamniotes where *myog*, the *mrf* that drives cell cycle exit and entry into terminal differentiation, is expressed before *pax7*; in amniotes, *Pax7* expression begins at a similar time point as *MyoG*. With the exception of *Six1*, trunk pre-myogenic genes are not expressed (e.g., *Pax3*) or not expressed consistently (e.g., *Eya1*). Moreover, head mesoderm genes have been shown to act directly upstream of *Mrf* (Zacharias et al., 2011; Moncaut et al., 2012; Castellanos et al., 2014). This suggests that in the phase before the onset of *Pax7*, the myogenic head mesoderm proceeds from a precursor state to a state where skeletal muscle formation is initiated, without recruiting the upstream factors controlling trunk myogenesis. This also suggests that in contrast to the trunk, head muscle precursor cells do not go through a phase of *Pax* gene expression before becoming a muscle stem cell.

### Head mesodermal cells may require a defined muscle environment to settle as muscle stem cells

When *Pax7* expression becomes detectable in the head mesoderm, the signal either occupies the same region as the *Mrf* signals or is nested within the *MyoD* expression domain. In turn, these markers overlap with the expression domains of the early head mesodermal genes. This indicates that in contrast to the early somite, the head mesoderm is not compartmentalized, and stem cells and differentiating cells emerge from within the same cell pool. This scenario is akin to the simultaneous renewal of stem cells and production of differentiating cells after -and from- *Pax7* expressing cells that have populated the myotome; the same occurs in the muscle masses of the limbs, and in all muscles during fetal and perinatal stages of development (reviewed in Buckingham and Vincent, 2009). Elegant studies in the mouse showed that both in the head and in the trunk, differentiating muscle displays the membrane-bound ligand Delta which triggers Notch signaling in the neighboring cells. This in turn suppresses *MyoD* expression and maintains the muscle stem cell state of these cells (Mourikis et al., 2012; Czajkowski et al., 2014; reviewed in Mourikis and Tajbakhsh, 2014). However, in the trunk, the initial expression of *Pax7* in the mouse dermomyotome is not controlled by a Notch-Delta lateral inhibition mechanism (Schuster-Gossler et al., 2007; Vasyutina et al., 2007). Similarly, the expression of *Pax7* in the head mesoderm was not Delta-dependent (Czajkowski et al., 2014). Thus, additional parameters have to be considered for the establishment of the head muscle stem cell pool.

### Head mesodermal cells may commit to myogenesis before becoming skeletal muscle stem cell

**Figure 14:**
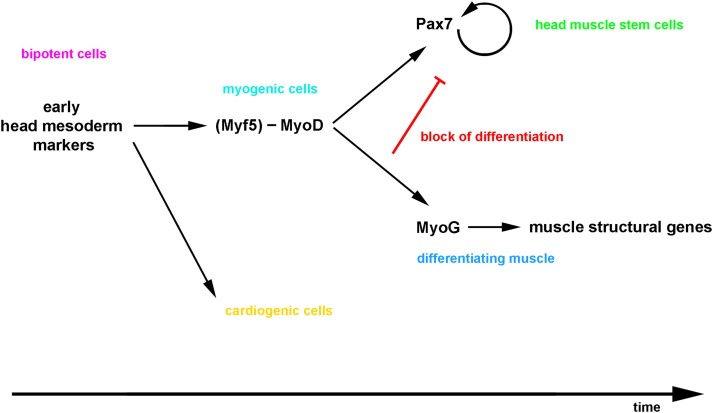
**Proposed model for the emergence of craniofacial muscle stem cells: the bi-potential head mesodermal cells commit to myogenesis before adapting a muscle stem cell state, and then a lateral inhibition mechanism initiated by the differentiating cells controls the simultaneous production of functional muscle and the maintenance of the stem cell pool**.

It is commonly held that at least in the amniote somite, all myogenic cells first express *Pax3* and *Pax7*, the *Pax* genes are genetically and molecularly upstream of *MyoD*, and when *MyoD* expression commences, the pre-myogenic genes are downregulated (reviewed in Buckingham and Vincent, 2009). The same observation has been made in satellite cells where, upon asymmetric cell division, the cell set up to activate *MyoD* will switch off *Pax7* and differentiate (Troy et al., 2012). Yet evidence is emerging that the linear progression from a *P*ax3/7+ state to a *M*yoD+ state is not obligatory: Lineage tracing and genetic cell ablations in the mouse have revealed that adult muscle stem cells have a history of *Myf5*, *MyoD* and *Mrf4* expression, indicating that the expression of Mrfs that control the initial myogenic commitment does not prevent the maintenance of a stem cell state (Kanisicak et al., 2009; Biressi et al., 2013; Sambasivan et al., 2013; Wood et al., 2013). In anamniotes, the first cells to form contractile muscle do not express *pax3/7* before undertaking myogenesis (reviewed in Bryson-Richardson and Currie, 2008), and in *Xenopus*, the *pax7* lineage is established in a zone lateral to the somite that also expresses *myoD* (Daughters et al., 2011; Della Gaspera et al., 2012). In the mouse myoblast cell line C2C12, quiescent stem cells arise concomitant with contractile cells when the cells are cultured differentiation promoting medium (Yoshida et al., 1998), and when *MyoD* is misexpressed in P19 embryonic carcinoma cells, the gene activates pre-myogenic rather than myogenic genes, and does so directly (Gianakopoulos et al., 2011). Thus, evidence is accumulating that *MyoD* can act upstream of *Pax7*. Our data showed that in the mouse, cells with current and with a history of *MyoD* expression are situated in the same territory and arise well before the onset of *Pax7*. Importantly, the majority of *Pax7* expressing cells develop from cells that previously expressed *MyoD*, and for the cells that do not display a history of *MyoD* expression, it cannot be excluded that thet expressed *Myf5* before. Interestingly, the closest chordate relatives of vertebrates, the ascidians, develop cardiac and pharyngeal muscles from a bi-potential precursor in a similar fashion to vertebrates (Stolfi et al., 2010; Wang et al., 2013). In these animals, the pharyngeal muscle stem cells express the single *mrf* gene before some cells are set aside to become stem cells (Razy-Krajka et al., 2014). Thus, while more detailed lineage tracing will be required to fully elucidate this question; our data suggests that vertebrate head mesodermal cells similarly proceed through a phase of *Mrf* gene expression which sets the stage for the activation of *Pax7* (a model is proposed in Figure 14).

### Myogenic commitment of head muscle stem cells may be obligatory and reflects the evolutionary history of the head mesoderm

Circulatory pumps equipped with contractile cells—hearts—are widespread in the animal kingdom, and a conserved, core regulatory network involving Nk4/tinman-type transcription factors may already have been established in Cnidarians (Shimizu and Fujisawa, 2003). Likewise, skeletal muscle for locomotion, generated with the help of a MyoD-like basic-helix-loop-helix transcription factor, is widespread and may predate the evolution of bilaterians (Muller et al., 2003, but see also Steinmetz et al., 2012). Yet, typically cardiac and skeletal muscle lineages are exclusive. Vertebrates and their closest chordate relatives, the ascidians, have evolved a program that generates cells for the heart as well as skeletal muscle. However, this muscle is not used for locomotion but is associated with the function of the pharynx. The muscularisation of the pharynx has been seen as a key step during vertebrate evolution as it provided the basis for the active ventilation of gills and eventually, the evolution of jaws (Gans and Northcutt, 1983). A central component in this system is the *Tbx1* gene (ascidians: single *Tbx1/10* gene; (Stolfi et al., 2010). In the more distantly related cephalochordate *Branchiostoma*, the *Tbx1/10* gene is expressed in the pharyngeal endoderm and mesoderm as well as the (ventral) somites (Mahadevan et al., 2004), while in the even further distant hemichordate *Saccoglossus kowalevskii Tbx1/10* is only found in the pharyngeal endoderm and gill slits (Gillis et al., 2012). This suggests that during evolution of the ascidian-vertebrate ancestor, *Tbx1/10* gene function has been linked both to the cardiac as well as the myogenic regulatory cascades. This implies that in contrast to the somite whose evolutionarily basic function is to generate skeletal muscle for locomotion, bi-potential pharyngeal cells have to commit to a myogenic fate before any muscle and muscle stem cells can be laid down. It is thus conceivable that, in the head, mesodermal cells have to express *Myf5, MyoD, or Mrf4* before they can be set aside as a muscle stem cell.

### Outlook

Our work provides the basis for the–testable—hypothesis that head mesodermal cells have commit to myogenesis, and once this is achieved, cells can faithfully execute standard myogenic programs. In line with this, we have observed that *Pax7* expressing head muscle stem cells provide the bulk of the head fetal muscles similar to muscle stem cells in the trunk (this study). Moreover, while the early head mesoderm is unable to provide muscle in a somitic environment (Mootoosamy and Dietrich, 2002), head muscle stem cells can shed their head mesodermal marker gene expression and regenerate trunk muscle (Sambasivan et al., 2009). Having established the emergence of head muscle stem cells, we can now explore the underlying molecular mechanisms and test, whether and in which environment head muscle stem cells can be redirected toward an earlier, bi-potential or a cardiogenic state.

### Conflict of interest statement

The authors declare that the research was conducted in the absence of any commercial or financial relationships that could be construed as a potential conflict of interest.
